# Neuroprotection of Botch in experimental intracerebral hemorrhage in rats

**DOI:** 10.18632/oncotarget.20524

**Published:** 2017-08-24

**Authors:** Binbin Mei, Haiying Li, Juehua Zhu, Junjie Yang, Ziying Yang, Zunjia Wen, Xiang Li, Haitao Shen, Meifen Shen, Gang Chen

**Affiliations:** ^1^ Department of Neurosurgery and Brain and Nerve Research Laboratory, The First Affiliated Hospital of Soochow University, Suzhou, Jiangsu Province, China; ^2^ Department of Neurology, The First Affiliated Hospital of Soochow University, Suzhou, Jiangsu Province, China; ^3^ Institute for Cardiovascular Science and Department of Cardiovascular Surgery of The First Affiliated Hospital, Soochow University, Suzhou, Jiangsu Province, China

**Keywords:** intracerebral hemorrhage, secondary brain injury, neuron, Botch, Notch1

## Abstract

Notch1 maturation participates in apoptosis and inflammation following intracerebral hemorrhage (ICH). It has been reported that Botch bound to and blocked Notch1 maturation. Here we estimated the role of Botch in ICH-induced secondary brain injury and underlying mechanisms. Experimental ICH model was induced by autologous arterial blood injection in Sprague-Dawley rats, and cultured primary rat cortical neurons were exposed to oxyhemoglobin to mimic ICH *in vitro*. Specific small interfering RNAs and expression plasmids encoding wild type Botch and Botch with Glu115Ala mutation were exploited. The protein levels of Botch and Notch1 transmembrane intracellular domain (Notch1-TMIC) were increased within brain tissue around hematoma. Botch overexpression led to an increase in unprocessed Notch1 full-length form accompanied by a significant decrease in Notch1-TMIC, while Botch knockdown resulted in an approximately 1.5-fold increase in Notch1-TMIC. There were increased cell apoptosis, necrosis and neurobehavioral deficits after ICH, which was inhibited by Botch overexpression and enhanced by Botch knockdown. Double immunofluorescence showed a colocalization of Botch and Notch1 in the trans-Golgi. Overexpression of wild type Botch, but not Botch E115A mutant, led to an increase in the interaction between Botch and Notch1, reduced the formation and the nuclear localization of Notch1 intracellular domain, and attenuated cell apoptosis and inflammation. In conclusion, Botch exerts neuroprotection against neuronal damage via antagonizing the maturation of Notch1 in Glu115-denpendent manner. However, neuroprotection mediated by endogenous Botch is not enough to reverse ICH-induced secondary brain injury.

## INTRODUCTION

As the second most common and deadliest type of stroke, intracerebral hemorrhage (ICH) is becoming an important public health problem [1, 2], which is associated with severe disability and high mortality. Although there is significant progress in clinical treatment, the outcomes of current treatment strategies for ICH are still not satisfying [3, 4].

The neuroprotective gene 7 (NPG7) is also known as Chac, cation transport regulator homolog 1, and is identified in functional screen for neuroprotective proteins [5]. Currently, the NPG7 is renamed Botch (Blocks Notch), which was widely expressed in multiple organs, including brain. Botch could prevent cell surface presentation of Notch1 by inhibiting the S1 furin-like cleavage of Notch1 and maintain Notch1 in the immature full-length form (Notch1-FL) [6]. The Notch signaling pathway is an evolutionarily conserved intercellular signaling pathway in most multicellular organisms [7]. As a member of Notch signaling pathway, there is increasing evidence that Notch1 signaling involves in neuropathological events including inflammatory central nervous system disease, brain and spinal cord trauma, ischemic stroke and hemorrhagic stroke [8–11]. Immature Notch1 is processed by cleavage by a furin-type protease to form a mature heterodimeric receptor in which one polypeptide becomes divided into the Notch1 extracellular domain (NECD) and the transmembrane intracellular domain (TMIC) [12–14]. The TMIC upon ligand binding to the NECD will undergo S2 and S3 cleavage to generate intracellular domain (NICD) [15, 16], which could translocate into the nucleus. In nucleus, NICD converts the C-promoter binding factor-1 (CBF-1) complex from a transcriptional repressor to a transcriptional activator resulting in expression of Notch target genes [16, 17], and then ultimately participate in differentiation, proliferation, apoptosis and inflammation [18–21].

These findings suggested that targeting Botch may provide novel insights into the suppression of the adverse reactions of Notch1 in neuropathological events. However, the relationship between Botch and Notch1 and the effect of Botch in ICH-induced secondary brain injury (SBI) remain obscure. Therefore, the aim of present study was to identify the roles of Botch and Notch1 in ICH-induced SBI in rats and the potential mechanisms, and assess the therapeutic potential of Botch following ICH.

## RESULTS

### ICH increased the protein levels of Botch and Notch1-TMIC in brain tissue around hematoma

**Figure 1:**
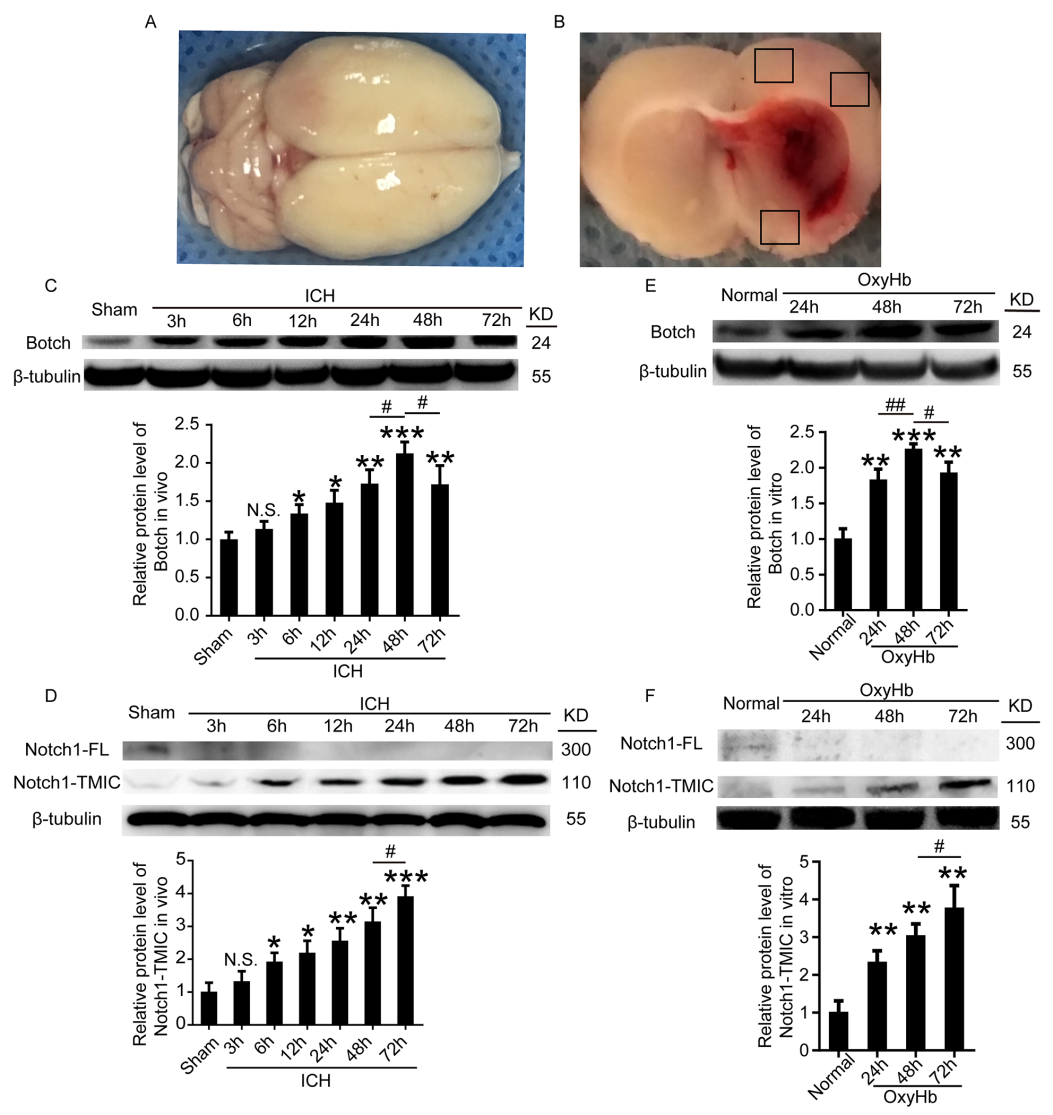
The protein levels of Botch and Notch1-TMIC in brain tissue around hematoma and cultured neurons following ICH and OxyHb treatment **(A)** Representative whole brains and autologous arterial blood injection ICH model. **(B)** Schematic representation of the suitable region taken for assay. **(C, D)** Western blot analysis and quantification of the protein levels of Botch and Notch1 in brain tissue around hematoma. **(E, F)** Western blots analysis and quantification of the protein levels of Botch and Notch1 in cultured neurons. In (C-F), mean values for sham or normal group were normalized to 1.0. (C) N.S. indicates no significant difference, p = 0.1609, 3 h vs sham; ^*^p = 0.0174, 6 h vs sham; ^*^p = 0.0113, 12 h vs sham; ^**^p = 0.0036, 24 h vs sham; ^***^p < 0.001, 48 h vs sham; ^**^p = 0.0088, 72 h vs sham; ^#^p = 0.0157, 48 h vs 24 h; ^#^p = 0.0142, 48 h vs 72 h. (D) N.S. indicates no significant difference, p = 0.2497, 3h vs sham; ^*^p = 0.0141, 6 h vs sham; ^*^p = 0.0106, 12 h vs sham; ^**^p = 0.0046, 24 h vs sham; ^**^p = 0.0018, 48 h vs sham; ^***^p < 0.001, 72 h vs sham; ^#^p = 0.0141,72 h vs 48 h. (E) ^**^p = 0.0019, 24h vs normal; ^***^p < 0.001, 48 h vs normal; ^**^p = 0.0013, 72 h vs normal; ^##^p = 0.0065, 48 h vs 24 h; ^#^p = 0.0248, 48 h vs 72 h. (F) ^**^p = 0.0047, 24 h vs normal; ^**^p = 0.0011, 48 h vs normal; ^**^p = 0.0018, 72 h vs normal; ^#^p = 0.0484, 72 h vs 48 h.

**Figure 2:**
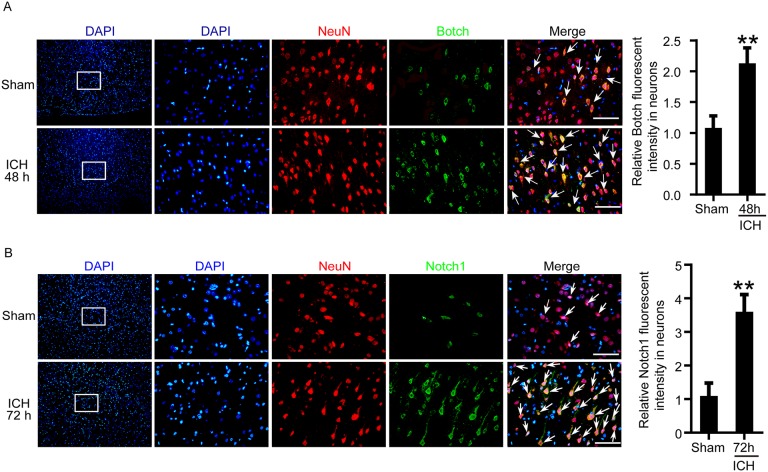
The protein levels of Botch and Notch1 in neurons around hematoma following ICH treatment **(A, B)** Double immunofluorescence analysis was performed with Botch/Notch1 antibodies (green) and neuronal marker (NeuN, red), and nuclei were fluorescently labeled with DAPI (blue). Arrows point to Botch/Notch1-positive neurons, scale bar = 100 μm. The relative fluorescent intensity of Botch/Notch1 in neurons was quantified. In (A, B), mean values for sham group were normalized to 1.0. (A) ^**^p = 0.0046, 48 h vs sham. (b) ^**^p = 0.0025, 72 h vs sham.

An experimental ICH model was established in Sprague–Dawley (SD) rats (Figure 1A and 1B). To detect the protein levels of Botch and Notch1in brain tissue around hematoma after ICH, a time course study was performed by western blot assay both *in vivo* and *in vitro*. The results demonstrated that the protein levels of Botch in brain tissue surrounding hematomas was significantly increased from 6 h after ICH and reached a peak level at 48 h (Figure 1C). And the level of Notch1-TMIC increased with time, peaked at 72 h, while Notch1-FL was only detected in sham group (Figure 1D). Similarly, the trend of the protein levels of Botch and Notch1 in cultured primary neurons *in vitro* was consistent with that *in vivo* data (Figure 1E and 1F). Additionally, double immunofluorescence assay further verified the ICH-induced increase in the protein levels of Botch and Notch1-TMIC in neurons (Figure 2A and 2B).

### Effects of overexpression and knockdown of Botch on the protein levels of Botch and the maturation of Notch1 in neurons under ICH insults both *in vivo* and *in vitro*

**Figure 3:**
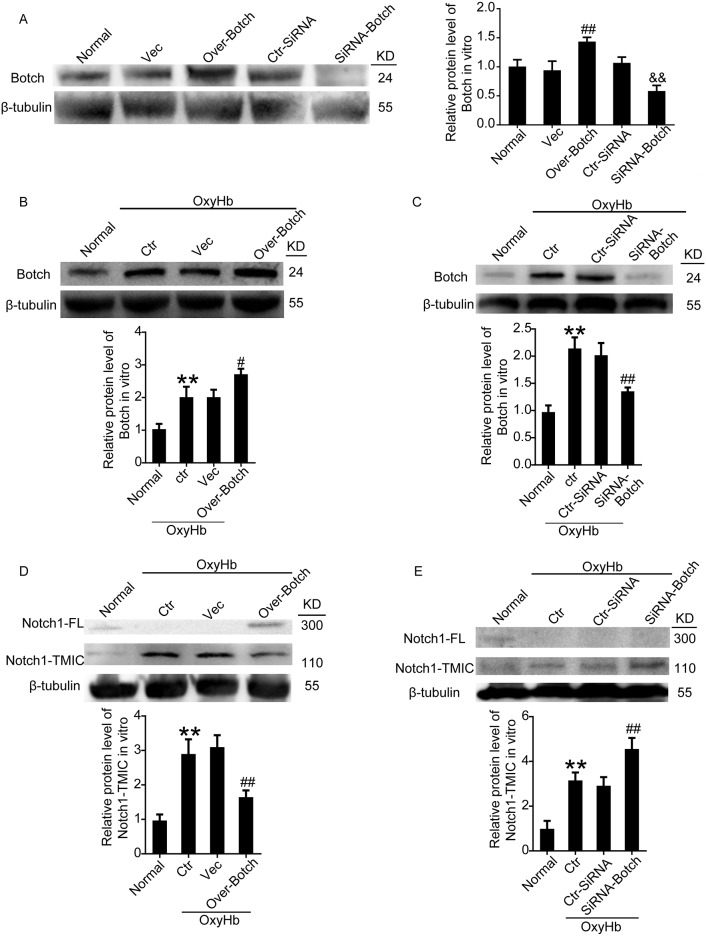
Effects of overexpression and knockdown of Botch on the protein levels of Botch and the maturation of Notch1 in cultured neurons **(A)** Transfection efficiency of Botch expression plasmid and siRNA in cultured neurons under normal condition. **(B, C)** Transfection efficiency of Botch expression plasmid and siRNA in cultured neurons exposed to OxyHb. **(D, E)** Effects of overexpression and knockdown of Botch on the protein levels of Notch1 in neurons under OxyHb insults. In (A-E), mean values for normal group were normalized to 1.0. (A) ^##^p = 0.0022 vs. vector; ^&&^ p = 0.0024 vs. ctr-siRNA. (B) ^**^p = 0.0090 vs normal; ^#^p = 0.0109 vs OxyHb + vector. (C) ^**^p = 0.0011 vs normal; N.S. indicates no significant difference; ^##^p = 0.0015 vs OxyHb + ctr-siRNA. (D) ^**^p = 0.0019 vs normal; ^##^p = 0.0032 vs OxyHb + vector. (E) ^**^p = 0.0017 vs normal; ^##^p=0.0021 vs OxyHb + ctr-siRNA.

**Figure 4:**
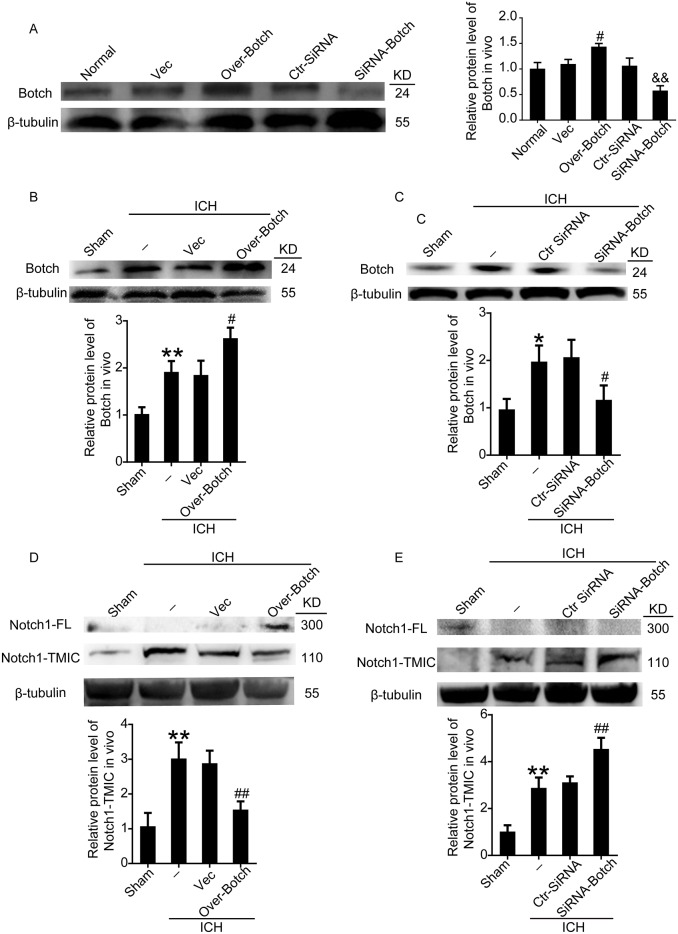
Effects of overexpression and knockdown of Botch on the protein levels of Botch and the maturation of Notch1 in brain tissue around hematoma **(A)** Transfection efficiency of Botch expression plasmid and siRNA in rat brains. **(B, C)** Transfection efficiency of Botch expression plasmid and siRNA in brains of ICH rats. **(D, E)** Effects of overexpression and knockdown of Botch on the protein levels of Notch1 in brain tissue under ICH insults. In (A-E), mean values for sham group were normalized to 1.0. (A) ^#^p = 0.0202 vs vector, ^&&^ p = 0.0017 vs ctr-siRNA. (B) ^**^p = 0.0039 vs sham, ^#^p = 0.0128 vs ICH + vector. (C) ^*^p = 0.0168 vs sham; ^#^p = 0.0305 vs ICH + ctr-siRNA. (D) ^**^p = 0.0050 vs sham; ^##^p = 0.0023 vs ICH + vector. (E) ^**^p = 0.0033 vs sham; ^##^p = 0.0032 vs ICH + ctr-siRNA.

To identify the effects of Botch on the maturation of Notch1, overexpression and knockdown of Botch by plasmid and siRNA transfection were implemented. The transfection efficiency of all the three siRNAs was verified by western blot analysis (data not shown), and the most efficient one (siRNA 2) was used in the following study. Western blot analysis further verified the efficiency of overexpression and knockdown of Botch in neurons both under normal condition (Figure 3A) and under OxyHb insults (Figure 3B and 3C). A contrasting result was appeared in the protein levels of Notch1: Botch overexpression led to a vastly increase in the levels of unprocessed Notch1-FL accompanied by a significant decrease in processed Notch1-TMIC (Figure 3D). And, Botch knockdown resulted in an approximately 1.5-fold increase in Notch1-TMIC (Figure 3E). Consistent with the *in vitro* data, the vivo western blot assay showed a similar trend (Figure 4).

### Effects of overexpression and knockdown of Botch on neuronal injury under ICH insults both *in vivo* and *in vitro*

**Figure 5:**
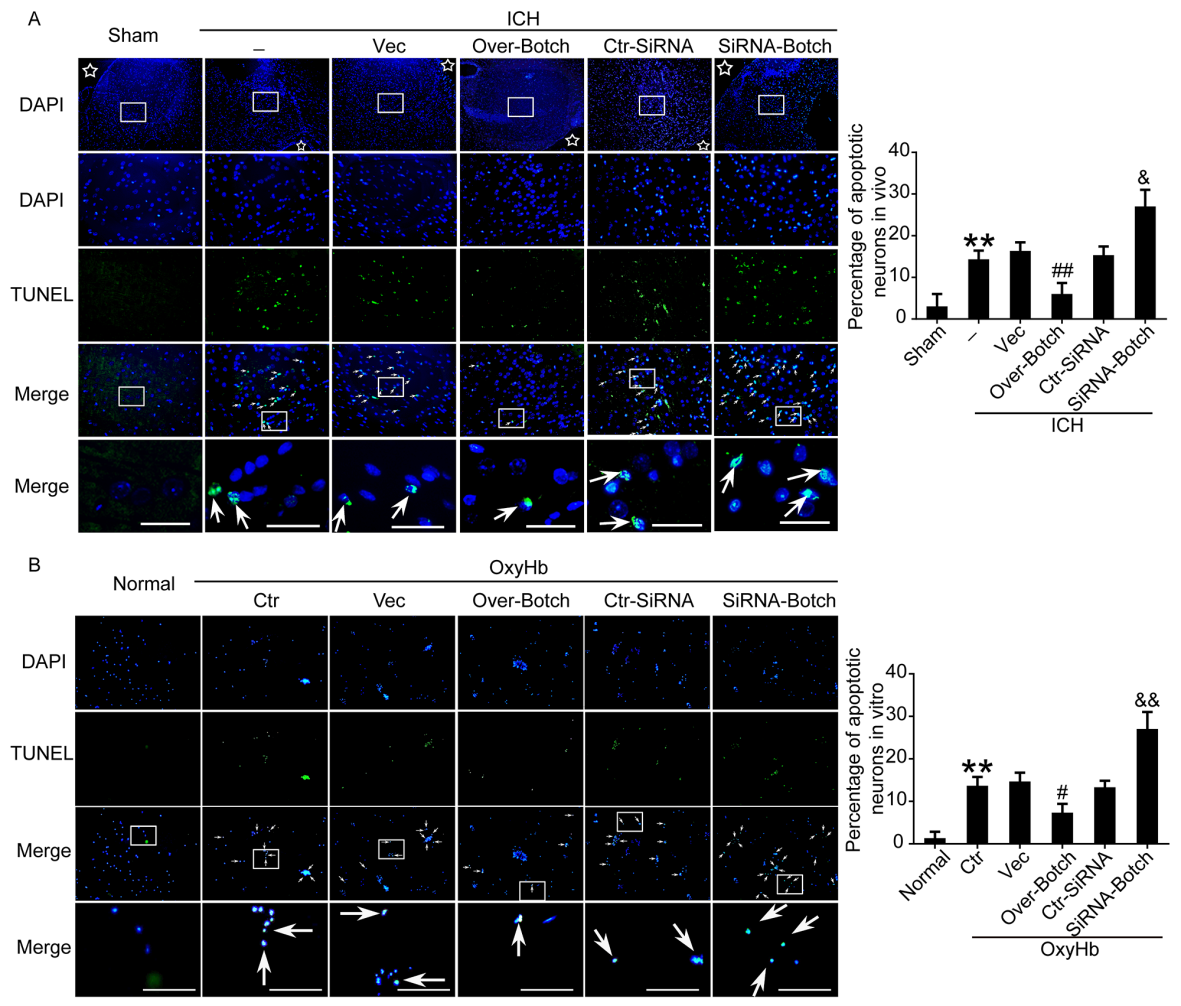
Effects of overexpression and knockdown of Botch on apoptosis in brain tissue around hematoma and cultured neurons exposed to OxyHb **(A)** Double staining for TUNEL (green) and DAPI (blue) *in vivo*. Arrows point to TUNEL-positive cells. Scale bar = 60 μm. Percentage of TUNEL-positive cells was shown. ^**^p = 0.0058 vs sham; ^##^p = 0.0060 vs ICH + vector; ^&^ p = 0.0110 vs ICH + ctr-siRNA. **(B)** Double staining for TUNEL (green) and DAPI (blue) *in vitro*. Arrows point to TUNEL-positive cells in cultured neurons. Scale bar = 100 μm. Percentage of TUNEL-positive cells in cultured neurons was shown. ^**^p = 0.0012 vs normal; ^#^p=0.0153 vs OxyHb + vector; ^&&^ p = 0.0052 vs OxyHb + ctr-siRNA.

**Figure 6:**
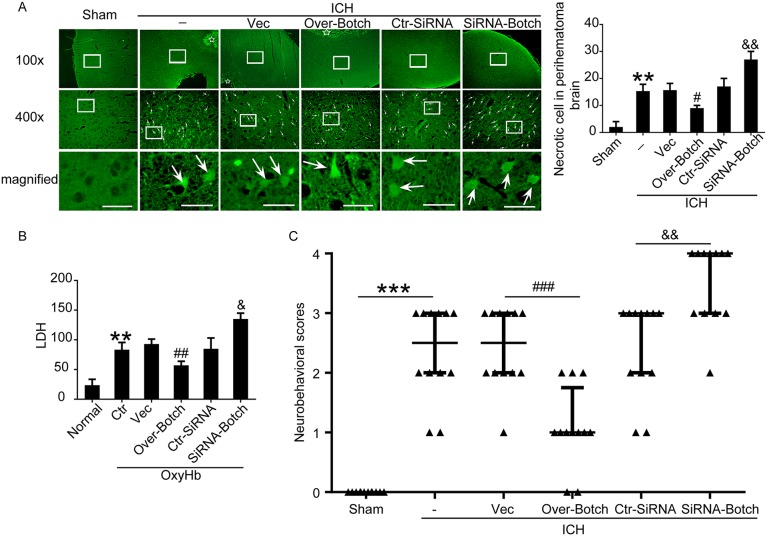
Effects of overexpression and knockdown of Botch on cell necrosis and neurological behavior under ICH and OxyHb insults **(A)** FJB staining showing the effects of Botch intervention on neuronal necrosis. Arrows point to FJB-positive cells in the brain. Scale bar = 60 μm. Quantification of the FJB staining as shown. ^**^p = 0.0020 vs sham; ^#^p = 0.0286 vs ICH + vector; ^&&^ p = 0.0015 vs ICH + ctr-siRNA. **(B)** LDH assay of cell culture supernatants. ^**^p = 0.0027 vs normal; ^##^p = 0.0071 vs OxyHb + vector; ^&^p = 0.0136 vs OxyHb + ctr-siRNA. **(C)** Neurological behavior scores. ^***^p < 0.0001; ^###^p = 0.0004; ^&&^ p = 0.0013.

To evaluate the effects of overexpression and knockdown of Botch on cell apoptotic and necrosis, terminal deoxynucleotidyl transferase dUTP nick end labeling (TUNEL) staining, fluoro-jade B (FJB) staining and lactate dehydrogenase (LDH) activity assay were performed both *in vivo* and *in vitro*. Compared with sham/normal group, a significant increase in the apoptotic index was observed in ICH/ OxyHb group, which was attenuated by Botch overexpression and exacerbated by Botch knockdown (Figure 5A and 5B). Similarly, FJB-positive cell in the brain samples significantly increased in the ICH group compared with sham group, which was significantly decreased by Botch overexpression and increased by Botch knockdown (Figure 6A). Consistently, LDH activity assay showed the same trend (Figure 6B).

### Effects of overexpression and knockdown of Botch on neurological behavior neurological behavior after experimental ICH

To identify the effects of Botch intervention on neurological behavior, behavioral activity was examined in all groups. Compared with the sham group, the rats after induction of ICH showed severe neurological behavior impairment, which was significantly alleviated with Botch overexpression treatment, yet more pronounced neurological defect was observed in Botch knockdown treatment group (Figure 6C).

### Botch antagonized the cleavage and maturation of Notch1 in Glu115 dependent manner

**Figure 7:**
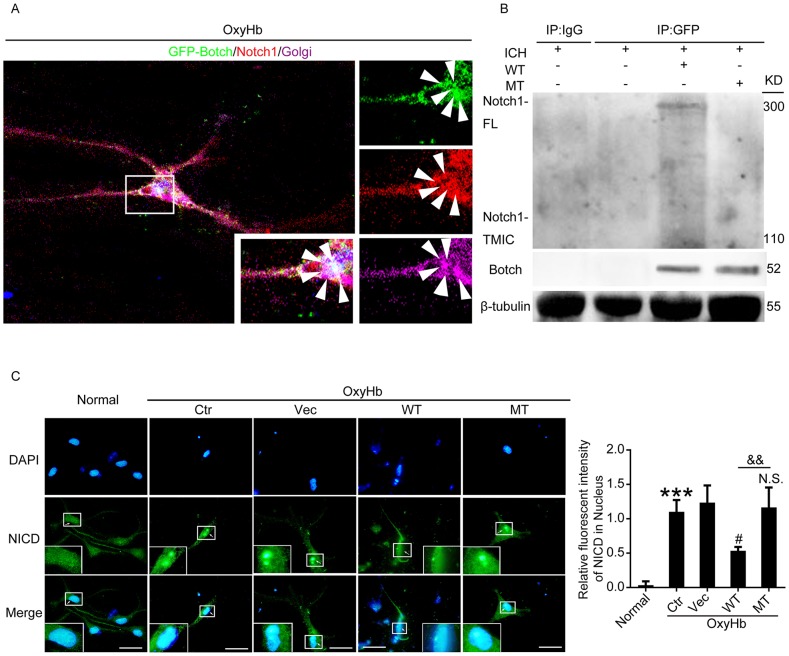
Botch antagonized the cleavage and maturation of Notch1 in Glu115 dependent manner **(A)** Multiple immunofluorescence for GFP-Botch (green) and Notch1 (red) counterstained with Golgi (purple) was performed under indicated treatment *in vitro*. Nuclei were fluorescently labeled with DAPI (blue). Arrows point to the colocations of Botch and Notch1 in Golgi. **(B)** Co-IP analysis of the interaction between GFP-Botch and Notch1 *in vivo*. **(C)** Immunofluorescence analysis was performed with NICD antibodies (green), and nuclei were fluorescently labeled with DAPI (blue). Scale bar = 20 μm. The relative fluorescent intensity of NICD in nucleus was shown. ^***^p < 0.001 vs normal; ^#^p = 0.0117 vs OxyHb + vector; N.S. indicates no significant difference vs OxyHb + vector; ^&&^ p = 0.0040.

To elucidate the underlying mechanisms of Botch-induced neuroprotection after ICH, expression vectors encoding wild type Botch and Botch with E115A mutation were prepared, and the immunofluorescence and co-immunoprecipitation (Co-IP) assay were performed. The vitro multiple immunofluorescence assay found that Botch and Notch1 colocalized within the trans-Golgi (Figure 7A). The *in vivo* co-immunoprecipitation (Co-IP) experiment showed that there was an interaction between wild type Botch and Notch1-FL, but not Notch-TMIC. Notably, E115A mutant almost completely inhibit the interaction between Botch and Notch1-FL (Figure 7B).

Immunofluorescence assay was implemented to describe the effects of Botch overexpression on the release of the NICD. Results showed that more NICD was released and translocated to the nucleus in the control group compared with the normal group, and wild type GFP-Botch overexpression decreased the levels of NICD in the nucleus, whereas E115A mutant GFP-Botch overexpression did not have the effect (Figure 7C).

### Effects of wide type Botch and mutant Botch overexpression on apoptosis and inflammation in cultured neurons exposed to OxyHb

**Figure 8:**
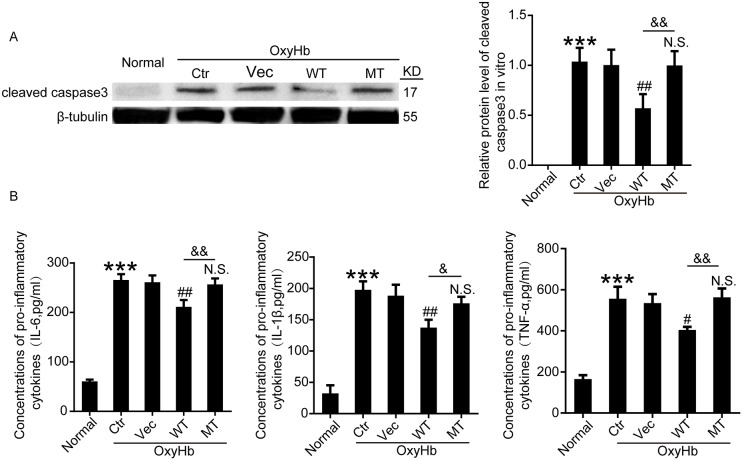
Effects of wide type Botch and mutant Botch overexpression on the cell apoptosis and inflammation in cultured neurons **(A)** The protein levels of cleaved caspase-3 were assessed by western blot analysis. ^***^p < 0.001 vs normal; ^##^p = 0.0064 vs OxyHb + vector; N.S. indicates no significant difference vs OxyHb + vector; ^&&^ p = 0.0023, OxyHb + WT vs OxyHb + MT. **(B)** Concentrations of pro-inflammatory cytokines (IL-6, IL-1β and TNF-α) in cell culture supernatants were assayed by ELISA. IL-6: ^***^p < 0.001 vs normal; ^##^p = 0.0019 vs OxyHb + vector; N.S. indicates no significant difference vs OxyHb + vector; ^&&^ p = 0.0037, OxyHb + WT vs OxyHb + MT. IL-1β: ^***^p < 0.001 vs normal; ^##^p = 0.0047 vs OxyHb + vector; N.S. indicates no significant difference vs OxyHb + vector; ^&^ p = 0.0271, OxyHb + WT vs OxyHb + MT. TNF-α: ^***^p < 0.001 vs normal; ^#^p = 0.0119 vs OxyHb + vector; N.S. indicates no significant difference vs. OxyHb + vector; ^&&^ p = 0.0031, OxyHb + WT vs OxyHb + MT.

To further ascertain the effects of wide type Botch and mutant Botch overexpression on apoptosis and inflammation, the protein levels of cleaved caspase-3 in neurons and the concentrations of IL-6, IL-1β and TNF-α in cell culture supernatant were detected by western blot analysis and enzyme-linked immunosorbent assay (ELISA), respectively. As shown in Figure 8A, wild type GFP-Botch overexpression suppressed the OxyHb-induced caspase-3 activation, however, this effect was abolished by E115A mutant. And we found that there were increased concentrations of IL-6, IL-1β and TNF-α in the culture supernatant of OxyHb group compared to normal group, which was significantly reduced by wild type GFP-Botch overexpression. However, GFP-Botch-E115A mutation overexpression did not have this effect (Figure 8B).

## DISCUSSION

Our present study showed the roles of Botch and Notch1 in the pathophysiological progression of SBI in a rat ICH model. First, results showed elevated levels of Botch and Notch1-TMIC in brain tissue around hematoma after ICH and cultured neuron exposed to OxyHb, while Notch1-FL was only detected in sham group. As the transmembrane and intracellular domain, Notch1-TMIC is a S1-cleaved mature form of Notch1 and is the dominant form of Notch1 [6]. Under ICH condition, the increased Notch1-TMIC and undetected Notch1-FL implied that Notch1 may be involved in the pathological process of ICH-induced SBI. Furthermore, there was an interaction between Botch and Notch1-FL, which could inhibit Notch1 mature in an E115-independent manner.

As previously reported [6, 22], Botch blocks Notch1 signaling through inhibition of the furin-like cleavage of Notch1 via its γ-glutamyl cyclotransferase (GGCT)-like activity, whereas Botch-E115A did not have this activity. So, E115 might be a key amino acid for Botch enzymatic function. As shown in Figure 7, Botch and Notch1 colocalized within the trans-Golgi, where Notch is processed by S1 cleavage by a furin-like protease [23]. Markedly, under ICH condition, wild type Botch interacted with the Notch1-FL, but not the Notch1-TMIC, while Botch E115A mutant lost this effect. The result was consistent with the previous research [6].

A series of secondary cascade of injury following ICH are referred to as SBI, the molecular and cellular mechanisms underlying the SBI are complicated, apoptosis and inflammation might play a momentous role in SBI pathology [19, 24, 25]. The activation of Notch1 results in the sequential proteolytic cleavage of the Notch1 receptor, which releases NICD into the nucleus. NICD in turn activates downstream gene transcription, eventually participates in the pathologic progress of SBI after ICH [26]. In our study, wild type Botch overexpression reduced the release and translocate to the nucleus of NICD, and attenuated cell apoptosis and inflammation, however, which was not statistically significant change with E115A mutant Botch overexpression. Noticeably, the variation tendency of NICD was consistent with inflammatory and apoptosis index, indicating further that the maturation of Notch1 participates in ICH-induced SBI, while Botch confers neuroprotection against SBI. Taken together, we further demonstrated that E115 was required for Botch to interferes with Notch1 maturation by interacting with Notch1.

**Figure 9:**
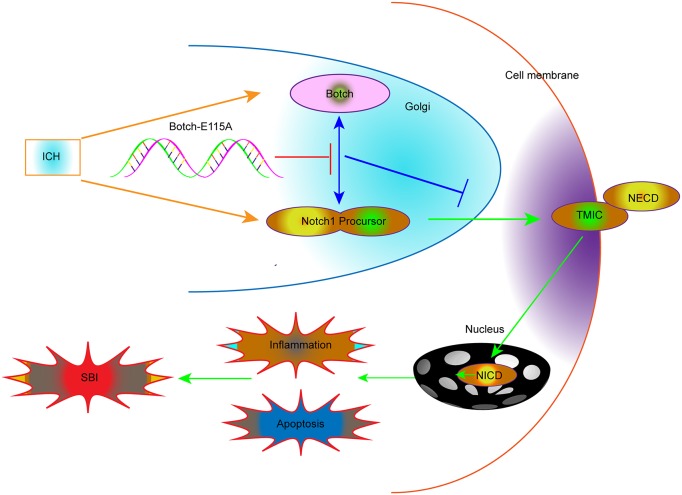
Schematic representations of the roles of Botch and Notch1 in SBI following ICH and the underlying mechanisms Briefly, Botch could significantly reduce ICH-induced SBI by effectively antagonizing Notch1, maintaining Notch1 in the immature full-length.

The inactive form of Notch1, before it reaching the plasma membrane, is firstly cleaved by a furin-like convertase in the trans-Golgi network to yield an active, ligand-accessible form. Ultimately, multiple cleavage processes result in the release and translocate to the nucleus of NICD, which subsequently activates transcription of downstream target genes and eventually involved in promoting cell apoptosis and inflammation following ICH. The cleavage and creation of the heterodimeric form of Notch1 occur in the Golgi apparatus, where Notch1 and Botch interact. Botch physical binds to the full-length unprocessed NECD interferes with the S1 furin-like cleavage of Notch1, whereas Botch-E115A is devoid of activity. Overall, as the negative regulator of ICH-induced SBI, Botch acts by maintaining Notch in an immature inactive form, which is dependent on the critical site E115. The mechanisms map is shown in Figure 9.

This study focused on the Botch and its possible role in neuroprotection following ICH. Botch, NPG7, was identified in functional screen for neuroprotective proteins, and there are previous studies on the correlation between Botch and the Notch1 signaling pathway [6, 22]. It is generally accepted that Notch1 plays a critical role in many fundamental processes and in a wide range of tissues, and it is not surprising that aberrant gain or loss of Notch1 signaling components has been directly linked to differentiation, proliferation, inflammation and apoptosis [13, 18–21, 27]. Yet, none of these studies have elucidated whether Botch could involve in the brain protection against ICH-induced SBI through its suppression effects of Notch1. In the current study, we found that Botch-induced neuroprotection was associated with the inhibition of Notch1 signaling and was dependent on the critical site E115. However, endogenous Botch was inadequate to completely reverse ICH-induced neuronal damage.

The current study has some limitations. In our experiment, we used healthy adult male SD rats, which did not mimic human high-risk populations maximally, such as patients with cardiovascular diseases and the elderly. And, different sex animals will be tested in our further study. Besides, the time course study showed that there was a difference in the peak time of protein levels of Botch and Notch1. We chose 48 h for following researches. However, whether there is other appropriate time to implement the intervention requires further investigation. Additionally, the remarkable rescue effect of Botch overexpression suggests a powerful therapeutic potential of Botch, however, how to better regulate the interaction between Botch and Notch1 is not answered and need to explore further.

In summary, this study showed a role of Botch in inducing neuroprotection against ICH-induced SBI for the first time, which is mediated through antagonize the cleavage and maturation of Notch1 in Glu115-dependent manner. And the remarkable alleviation in the cell apoptosis and inflammation after Botch overexpression, suggesting that therapies targeting Notch1 hold significant promise for the treatment and prevention of pathologic processes characterized by ICH-induced brain injury, and Botch might be good target for improving SBI after ICH. However, neuroprotection mediated by endogenous Botch is not enough to reverse ICH-induced secondary brain injury.

## MATERIALS AND METHODS

### Animals

The experiments were approved by the Ethics Committee of the First Affiliated Hospital of Soochow University and were implemented in strict accordance with the guidelines of the National Institutes of Health on the care and use of animals. All adult male SD rats weighing between 250 and 300 g were purchased from Animal Center of Chinese Academy of Sciences, Shanghai, China. The rats were housed in temperature- and humidity-controlled animal quarters with a 12-h light/dark cycle.

### Rat ICH model

Experimental ICH model was a modification of the method as described previously [28, 29]. Firstly, rats were anesthetized with 4% chloral hydrate (10 ml/kg, Intraperitoneal injection), and fixed in the prone position to a stereotactic frame (Shanghai Ruanlong Science and Technology Development Co., Ltd., Shanghai, China). Rats were positioned with the anterior and posterior fontanelles in the same horizontal plane. The median scalp was shaved and sterilized, and a median incision approximately 1 cm long was made. The periosteum was stripped using a bone stripper, and the anterior fontanelle and coronal suture exposed. A round hole (1.0 mm diameter) was drilled using a dental drill (3.5 mm right and 0.2 mm posterior to bregma) until the dural surface was reached. 80 ul non-heparinized autologous arterial blood were collected by cardiac puncture and slowly injection at a rate of 16ul/min using a microinjector fixed on the stereotaxic apparatus, which entered vertically approximately 5.5 mm along the hole, and maintained in place for 5 min. Finally, the needle was removed and the scalp sutured. A schematic representation of the brain coronal sections for assay was shown in Figure 1A and 1B.

### Cell culture

Primary rat cortical neurons were obtained and cultured as described previously [30]. Briefly, cortical neurons were isolated from E16-18 rat embryos, and treated with Trypsin-EDTA Solution for 5 min at 37°C. Dissociated neurons were plated onto plates (Corning, USA) precoated with 0.1 mg/ml poly-D-lysine (Sigma, USA), cultured in Neurobasal-A medium supplemented with 2% B-27 and 0.5 mM GlutaMAX™-I (all from Invitrogen, Grand Island, NY), and maintained at 37°C under humidified conditions and 5% CO2 for approximately 2 weeks. Half of the media were exchanged for fresh media every two days.

### Experimental design

The total experiments were composed of two sections, and each part all included both *in vivo* and *in vitro* experiments. In experiment 1, 42 rats (50 rats were used, 42 rats were survived after the surgery) were randomly assigned to 7 groups of 6 rats each, a sham group, and 6 experimental groups arranged by time: 3, 6, 12, 24, 48 and 72 h after ICH. Then brain tissue samples were obtained separately from rats for the time course study for the protein levels of the Botch and Notch1. Then, to simulate ICH *in vitro*, enriched neurons were divided into 4 groups: normal, 24, 48, 72 h after exposed to OxyHb.

Based on the results of the time course study, 48h after ICH or OxyHb treatment was exploited in experiment 2. First, another 84 rats were randomly divided into 7 groups: sham, ICH, ICH + vector, ICH + wild type Botch overexpression (WT), ICH + Botch E115A overexpression (MT), ICH + control-siRNA (ctr-siRNA), ICH + siRNA-Botch. The brain cortex of rats was extracted for TUNEL staining, FJB staining, Co-IP and western blot analysis for the roles of Botch in ICH-induced SBI and the potential mechanisms. Similarly, to explore the roles of Botch *in vitro*, neurons were divided into 7 groups: normal, OxyHb + control, OxyHb + vector, OxyHb + Botch-WT, OxyHb + Botch-MT, OxyHb + ctr-siRNA, OxyHb + siRNA-Botch. After these treatments, the total protein of the cells was collected and stored at -80 °C until tested, and cells for immunofluorescence analysis were fixed with 4 % paraformaldehyde.

### Antibodies

Notch1 antibody (ab27526), Chac1 antibody (ab76386), Rb pAb to activated Notch1 (ab52301), Rb mAb to NeuN (ab177487), Ms mAb to NeuN (ab104224) were from Abcam. β-tubulin (sc-9014), TGN 38 (sc-27680), normal rabbit IgG (sc-2027), normal mouse IgG (sc-2025) and normal goat IgG (sc-2028) were from Santa Cruz Biotechnology. Anti-Botch1/Chac1 (75-181) was from Neuromab. Cleaved caspase-3 (D175) was from Cell Signaling Technology. Protein A+G Agarose (P2012) was from Beyotime. Secondary antibodies for western blot analysis, including goat anti-rabbit IgG-HRP (sc-2004), donkey anti-goat IgG-HRP (sc-2020), and goat anti-mouse IgG-HRP(sc-2005) were from Santa Cruz Biotechnology. Secondary antibodies for immunofluorescence, including Alexa Fluor-488 donkey anti-rabbit IgG antibody (A21206), Alexa Fluor-488 donkey anti-mouse IgG antibody (A21202), Alexa Fluor-555 donkey anti-mouse IgG antibody (A31570), Alexa Fluor-555 donkey anti-rabbit IgG antibody (A31572), and Alexa Fluor-633 donkey anti-goat IgG antibody (A21082) were from life technologies.

### Construction of expression plasmids and site directed mutagenesis

Specific expression plasmid of Botch was obtained from Ribobio. For immunofluorescence analysis, the coding region of rat Botch cDNA was sub-cloned into pEGFP-N2 expression vector to produce the pEGFPN2-Botch construct. In addition, a rat Botch cDNA construct with a mutation in a possible key site (Glu115) was prepared. E115A mutant (Glu115 of Botch was mutated to Ala) was also sub-cloned into the pEGFP-N2 expression vector as the wild-type Botch cDNA, which allowed us to measure their location by fluorescence assay. All of the constructs were confirmed by DNA sequencing.

### Plasmid transfection in rat brain

The vivo plasmid transfection in rat brain was performed according to the manufacturer's instructions for Entranster-*in vivo* DNA transfection reagent (Engreen, 18668-11-2). Firstly, 10 μL Entranster-*in vivo* DNA transfection reagent was added to 5 μL plasmid or 5 μL empty vector immediately. The solution was mixed for 15 min at room temperature. Finally, 15 μL Entranster-*in vivo*-plasmid mixture was injected intracerebroventricularly at 48 h before ICH.

### Transfection of siRNA in rat brain

According to the manufacturer's instruction for Entranster-*in vivo* RNA transfection reagent (Engreen, 18668-11-1), the transfection complex of siRNA was prepared as follows. Briefly, 5 nmol Botch siRNA and 5 nmol scramble siRNA were respectively dissolved in 66.5 μL DEPC RNase-free water. Then, 5 μL Entranster-*in vivo* RNA transfection reagent and 5 ul normal saline were added to 10 μL siRNA or 10 μL scramble siRNA immediately. The solution was mixed for 15 min at room temperature. Finally, 20 μL Entranster-*in vivo*-siRNA mixture was injected intracerebroventricularly at 48 h before ICH.

Specific siRNAs against Botch were obtained from Ribobio. The knockdown efficiency of all the three siRNAs was separately tested by vitro western blot analysis. The most efficient one was used in the following study.

Botch siRNA sequences:

1. Sense: 5′GAGAGAAGCUGUGCUUGGU dTdT 3′

Antisense: 3′dTdT CUCUCUUCGACACGAACCA 5′

2. Sense: 5′CACUGAAGUACCUGAACGU dTdT 3′

Antisense: 3′dTdT GUGACUUCAUGGACUUGCA 5′

3. Sense: 5′CUAAGGAAGUCACCUUUUA dTdT 3′

Antisense: 3′dTdT GAUUCCUUCAGUGGAAAAU 5′

### Transfection of plasmid and siRNA in cultured neurons

Cultured neurons were transfected with indicated expression vectors using Lipofectamine® 3000 Transfection Reagent (Invitrogen, L3000-015) or siRNAs using Lipofectamine RNAi MAX (Invitrogen, 13778-075) according to the manufacturer's instructions. At 48 h after transfection, neurons were treated with OxyHb for an additional 48 h. Then neurons were harvested for further analysis.

### Neurological impairment

The effects of Botch intervention on behavioral impairment were examined using a previously published scoring system and monitored for appetite, activity, and neurological defects [31].

### Western blot analysis

*In vivo*, cortices were sampled 1 mm away from the hematoma to avoid potential red blood cell contamination. The brain samples and cells were collected and lysed in ice-cold RIPA lysis buffer (P0013; Beyotime, Shanghai, China). After centrifuge at 12,000 g for 10 min at 4°C, the supernatants were collected. The protein concentration was measured using the bicinchoninic acid kit (Beyotime, P0010). The protein samples were loaded on SDS polyacrylamide gel, separated, and electrophoretically transferred to a polyvinylidene difluoride membrane (IPVH00010; Millipore, Billerica, MA, USA). The membrane was blocked with 5% non-fat milk for 2 h at room temperature. Subsequently, the membrane was probed with the primary antibody against Botch, Notch1, and cleaved caspase-3 overnight at 4°C. The β- tubulin was used as a loading control. Then they were incubated in the appropriate HRP-conjugated secondary antibodies for 1.5 h at room temperature and washed with PBST. Finally, the protein bands were visualized using enhanced chemiluminescence. The relative quantity of proteins was analyzed using Image J and normalized to that of the loading control.

### Immunofluorescence analysis

The vivo immunofluorescence analysis was prepared as follows. Briefly, the brain samples were fixed in 4% paraformaldehyde, embedded in paraffin, cut into 4 μm sections which were stained with primary antibodies (diluted 1:100) and appropriate secondary antibodies (1:500dilution). Normal rabbit IgG or normal mouse IgG was used as a negative control (data not shown). Finally, sections were observed by a fluorescence microscope (OLYMPUS BX50/BX-FLA/DP70; Olympus Co., Japan), and the relative fluorescence intensity was analyzed by use of Image J program.

The *in vitro* multiple labeling immunofluorescence analysis was also performed. In brief, cells were fixed in 4 % paraformaldehyde for 15 min at room temperature and blocked in 5 % bovine serum albumin (Biosharp, Hefei, China) for 30 min, then incubated with primary antibody overnight at 4 °C. Then, cells were washed with PBST for three times and incubated with another antibody overnight at 4 °C. Repeated the above process until the total primary antibodies all used. Finally, cells were washed with PBST and incubated with corresponding appropriate secondary antibodies at 37 °C for 30-60 min. The fluorescence images were captured using a laser scanning confocal microscope (ZEISS LSM 880, Carl Zeiss AG, Germany).

### TUNEL staining

Cell apoptosis in brain tissue was detected by TUNEL staining according to the manufacturer's protocol (DeadEnd Flurometric kit, Promega, WI, USA). Briefly, brain tissues were paraffin embedded and sectioned, and then heated and dewaxed. After dewaxed, the sections were incubated in Triton X-100 for 8 min. Then the sections were washed 3 times with PBS (5 min per wash), and then incubated with TUNEL-staining at 37 °C for 1 h. Nuclei were stained with DAPI (Southern Biotech, Birmingham, AL, U.S.) mounting medium after washed 3 times with PBS at room temperature. Finally, the sections were visualized by a fluorescence microscope (OLYMPUS BX50/BX-FLA/DP70; Olympus Co., Japan.). To explore the extent of cell apoptosis, the apoptotic index was defined as the percentage of TUNEL-positive cells in each section. Cell apoptosis in enriched neurons was also examined by TUNEL staining and the procedure was similar to the above process, excluding the neurons was fixed in 4% paraformaldehyd, and incubated in Triton X-100 for 2 min.

### FJB staining

Cell necrosis in brain tissue was detected by FJB staining. Brain sections were deparaffinized, dehydrated, incubated in 0.06% K permanganate for 10 min, then rinsed in deionized water and immersed in FJB working solution (0.1% acetic acid) for 20 min and dried in an incubator (50-60 °C) for 10 min. Sections were cleared in xylene and cover slipped with a non-aqueous, low-fluorescence, styrene-based mounting medium(DPX, Sigma-Aldrich, MO, U.S.). The sections were visualized by a fluorescence microscope (OLYMPUS BX50/BX-FLA/DP70; Olympus Co., Japan). The number of FJB-positive cells in each section was calculated carefully per sample, and cell counts from the section were averaged to provide the mean value.

### Assay of LDH activity

The level of LDH in cell culture supernatant was quantified using LDH kit following the manufacturers’ instruction (Nanjing Jiancheng Bioengineering Institute, Nanjing, China). Firstly, the reaction wells included standard wells, sample wells, control wells and empty wells were created, and enough reagents for the number of assays were prepared. Then the reaction mix was performed and added into the relative groups. Finally, the activity of LDH was measured immediately at OD 450 nm on a microplate reader.

### Co-immunoprecipitation (Co-IP) analysis

Co-IP analysis was performed as described previously [32]. Firstly, the brain samples were lysed in the ice-cold RIPA lysis buffer. And the lysate was incubated with specific antibodies or normal IgG (negative control) for 1 h at 4 °C with agitation. Then protein A+G agarose beads were added to each immune complex and the lysate-bead mixture was incubated overnight at 4°C with rotary agitation. SDS-PAGE and immunoblotting were then performed for further protein separation and detection.

### Assay of inflammatory cytokines

The levels ofIL-6, IL-1β and TNF-α (Cloud-Clone Corp.) in cell culture supernatant were quantified using specific ELISA kits for rats according to the manufacturers’ instructions. Firstly, determined wells for diluted standard, blank and sample, and standard, blank and samples were added to the plates and incubated for 1 h at 37 °C. Next, detection reagent was added to the each well and incubated for 30min, followed by 90ul substrate solution. Then reaction was stopped by adding 50ul of stop solution. Finally, ran the microplate reader and conducted measurement at 450 nm immediately.

### Statistical analysis

Graphpad prism 6 was used for all statistical analysis. Neurobehavioral scores were shown as median with interquartile range. Frequency distribution for the neurobehavioral score assay. The Mann-Whitney U test was used to compare behavior and activity scores among groups.

All the other data are presented as mean ± SD. Data were analyzed by one-way ANOVA followed by either a Dunnett's or a Sidak's post hoc test, the former for comparisons to a single control group, the latter for comparisons with the preselected pairs of groups, P< 0.05 was considered statistically significant.

## Abbreviations

ICH: intracerebral hemorrhage
SBI: secondary brain injury
SD: Sprague-Dawley
OxyHb: oxyhemoglobin
Notch1-FL: Notch1 full-length form
NECD: Notch1 extracellular domain
Noch1-TMIC: Notch1 transmembrane intracellular domain
NICD: Notch1 intracellular domain
NPG7: neuroprotective gene 7
CBF-1: C-promoter binding factor-1
TUNEL: Terminal Deoxynucleotidyl Transferase dUTP Nick End Labeling
FJB: Fluoro-Jade B
LDH: Lactate Dehydrogenase
GGCT: γ-glutamyl cyclotransferase
Co-IP: Co-immunoprecipitation
WT: wide type GFP-Botch
MT: E115A mutant GFP-Botch
